# Apigenin Attenuates Melanoma Cell Migration by Inducing Anoikis through Integrin and Focal Adhesion Kinase Inhibition

**DOI:** 10.3390/molecules201219752

**Published:** 2015-11-27

**Authors:** Md. Abul Hasnat, Mehnaz Pervin, Ji Hong Lim, Beong Ou Lim

**Affiliations:** Department of Life Sciences, College of Biomedical & Health Science, Konkuk University, Chungju 380-701, Korea; noman33ju@gmail.com (M.A.H.); mehnaz.pervin@gmail.com (M.P.)

**Keywords:** apigenin, melanoma, anoikis, migration, integrin-FAK signaling

## Abstract

Apigenin, a nonmutagenic flavonoid, has been found to have antitumor properties and is therefore particularly relevant for the development of chemotherapeutic agents for cancers. In this study, time- and dose-dependent cell viability and cytotoxicity were assessed to determine the effects of apigenin on A2058 and A375 melanoma cells. Melanoma cells were pretreated with different concentrations of apigenin and analyzed for morphological changes, anoikis induction, cell migration, and levels of proteins associated with apoptosis. Apigenin reduced integrin protein levels and inhibited the phosphorylation of focal adhesion kinase (FAK) and extracellular signal-regulated kinase (ERK1/2), which induce anoikis in human cutaneous melanoma cells. Apigenin exhibited dose-dependent inhibition of melanoma cell migration, unlike untreated controls. Furthermore, apigenin treatment increased apoptotic factors such as caspase-3 and cleaved poly(ADP-ribose) polymerase in a dose-dependent manner, demonstrating the metastasis of melanoma cells. Our results provide a new insight into the mechanisms by which apigenin prevents melanoma metastasis by sensitizing anoikis induced by the loss of integrin proteins in the FAK/ERK1/2 signaling pathway. These findings elucidate the related mechanisms and suggest the potential of apigenin in developing clinical treatment strategies against malignant melanoma.

## 1. Introduction

Melanoma is the most aggressive form of skin cancer, causing more than 90% of the deaths resulting from this disease [1]. Early-stage primary melanoma is curable by surgery, but the treatment of late-stage metastatic melanoma is very difficult. Most standard chemotherapy cancer drugs for this tumor have not cleared large-scale clinical trials. Multiple intracellular signaling pathways targeted by small-molecules are a known strategy in melanoma therapeutics [2]. Searching for effective drugs to treat metastatic melanoma is a challenging task because of the strong recalcitrance of melanoma to drugs.

Metastasis is a multistep and complex process. It involves the detachment of carcinoma cells from the originated sites, degradation of the extracellular matrix (ECM), survival in the vascular system, invasion of surrounding tissues, cell adhesion, and proliferation in distant sites in the body [3]. Cells usually go through an apoptotic process known as “anoikis” to prevent metastasis. The word “anoikis” was first used by Frisch and Francis in 1994 when they noticed an apoptosis in epithelial cells due to loss of appropriate matrix attachment [4]. Cancer cells acquire anoikis resistance to survive after detachment from the primary sites. Anoikis or detachment-induced apoptosis resistance and anchorage independence allow cancer cells to expand and invade adjacent tissues and to disseminate through the body, giving rise to metastasis.

Different steps in the metastatic process are associated with integrins and it is shown by the ubiquitous presence of integrins in blood components, vasculature, tumor and stromal cells. Integrins are a family of heterodimeric (αβ) transmembrane receptors that mediate both cell–ECM and cell–cell adhesion. Various types of intracellular signaling processes, such as proliferation, differentiation, invasion, metastasis, apoptosis, and anoikis are mostly influenced by Integrins [5,6,7]. Progression of malignant melanoma is linked with an altered expression of a number of integrins [8]. The integrin family of transmembrane receptors link the ECM to the intracellular actin cytoskeleton at points of cell–substratum interaction termed focal adhesions.

Focal adhesion kinase (FAK) is a cytoplasmic tyrosine kinase activated after integrin-mediated adhesion to ECM proteins, and it is involved in integrin-mediated signal transduction pathways [9,10]. In adherent cells, FAK colocalizes with integrins at points of focal contact. In different variety of cells, FAK activation and tyrosine phosphorylation have found to depend on integrins binding to their extracellular ligands [10]. FAK expression is required for many normal cellular functions in development and angiogenesis, but its expression is upregulated in a variety of late-stage cancers. Increased levels of FAK in 17 invasive colon tumors and in 15 metastatic tumors were reported by Weiner *et al.* [11]. FAK is most likely involved in extracellular signal-regulated protein kinase (ERK)-mediated cell migration. ERK1/2, a subfamily of the mitogen-activated protein kinases (MAPKs), is one of the best characterized intracellular signaling pathways, which plays a crucial role in regulating the invasion and metastasis of melanoma [12]. ERK1/2 is involved in cell death determination, tumor progression, angiogenesis, and metastasis. Inhibition of ERK1/2 has been found to reduce the metastatic potential of melanoma cells [13]. Caspase-3, a member of the caspase family plays a central role in the execution of the apoptotic program. Cleavage of poly(ADP-ribose) polymerase (PARP) by proteolytically active caspase-3 is considered to be a hallmark of apoptosis [14]. Cleavage of caspase-3 and PARP induces anoikis in melanoma cells [15].

Apigenin (4′,5,7,-trihydroxyflavone), a nonmutagenic and low-toxicity dietary flavonoid abundantly present in many fruits and vegetables, including parsley, onions, orange, tea, chamomile, wheat sprouts, and in some seasonings, has a broad spectrum of antiproliferative activities against many types of cancer cells [16,17]. Recent studies have demonstrated that apigenin inhibits cancer cell growth through cell cycle arrest and apoptosis in malignant human cancer cell lines [18,19]. Apigenin suppresses angiogenesis in melanoma and carcinoma of the breast, skin, and colon [20,21,22,23]. Apigenin was also used with poly(lactic-co-glycolide) nanoparticles to prevent skin tumors induced by ultraviolet B (UVB) radiation and benzo(a)pyrene (BaP) treatment in mice [24]. Apigenin potentially inhibits epidermal growth factor receptor and tyrosine kinase [25,26]. Previous reports also showed that apigenin successfully modulate the expression of different upstream kinases which are involved in the development and progression of cancer [27,28,29].

Although apigenin has been found to possess antitumor properties in many studies, the underlying mechanisms by which this compound inhibits cancers are not understood. In the present study, we sought to investigate the effect of apigenin on the proliferation of melanoma cells. We report that apigenin induces anoikis, a type of apoptosis induced by the loss of integrin-mediated cell matrix contact. We also tried to explain the possible molecular mechanisms involved in the process.

## 2. Results

### 2.1. Apigenin Inhibits Proliferation and Viability of Human Melanoma Cells and Induces Anoikis

**Figure 1:**
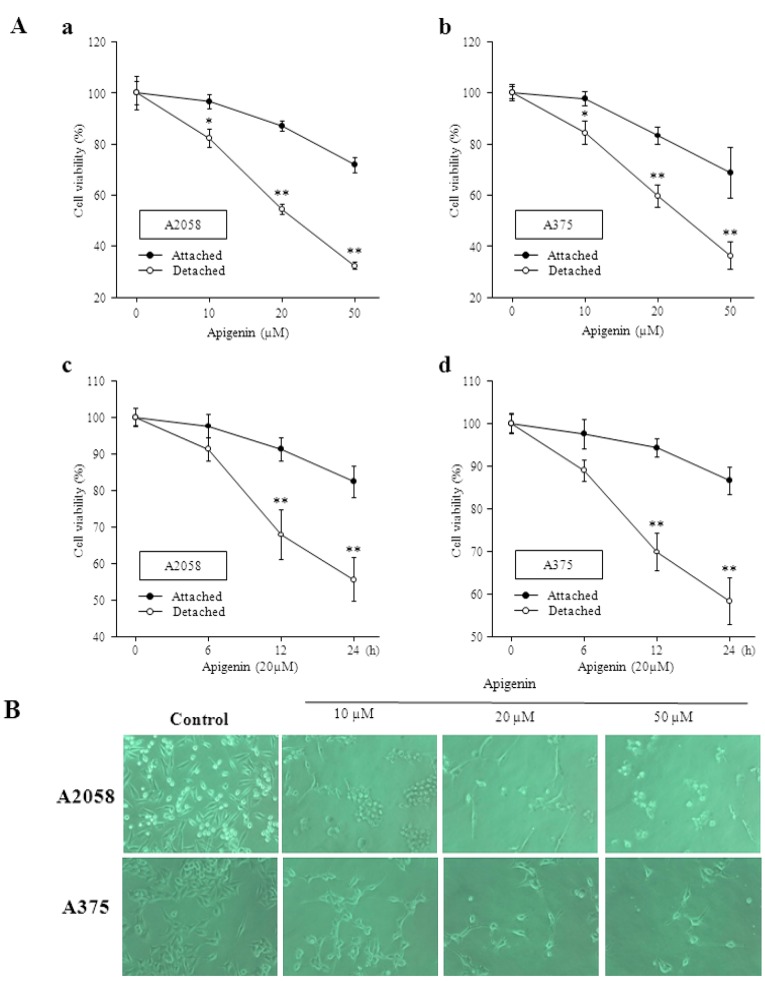
Apigenin inhibits proliferation and viability of human melanoma cells and induces anoikis. (**A**) Cell viability was quantified by MTT assay in attached and detached condition. A2058 and A375 cells were treated with different concentrations of apigenin for 24 h (a and b) and for different time intervals (c and d) at 37 °C; * and ** Indicates significant differences (*p* < 0.05) compared to the untreated control (**B**) A375 and A2058 cells were left untreated or were treated with apigenin at different concentrations for 24 h and morphological changes of the cells were observed by light microscopy and photographed at a magnification of 100×.

To investigate the anticancer effects of apigenin, human cutaneous melanoma cells (A2058 and A375) were treated with various concentrations of apigenin for different time intervals, and the numbers of viable cells remaining were assessed in both attached and detached conditions. In both conditions, apigenin treatment showed significantly decreased proliferation of cells in dose-dependent and time-dependent manners (Figure 1A). According to MTT assay results, treatment with 50 µM apigenin significantly reduced viable cell percentages in both types of melanoma cells. Treatment with apigenin for 24 h also decreased human melanoma cell numbers in a dose-dependent manner, as illustrated by cell morphology assays. Under light microscopy, apigenin-treated cells exhibited a rounded and granulated morphology and eventually degraded after treatment with up to 50 µM apigenin (Figure 1B). Irregular morphological shape and decreased proliferation, especially in detached condition indicates induction of anoikis. To investigate the influence of apigenin on normal cells, we examined the cell viability in apigenin-treated Raw 264.7 macrophage cells by MTT assay. Compared to the control (DMSO-treated alone), there was no significant difference in cell viability at concentrations ranging from 0 to 50 µM (data not shown). This indicated that apigenin inhibited cell proliferation, induced anoikis, and caused cell death in melanoma cells but did not have a detrimental effect on normal cells.

### 2.2. Inhibitory Effect of Apigenin on Integrin Protein Expression

**Figure 2:**
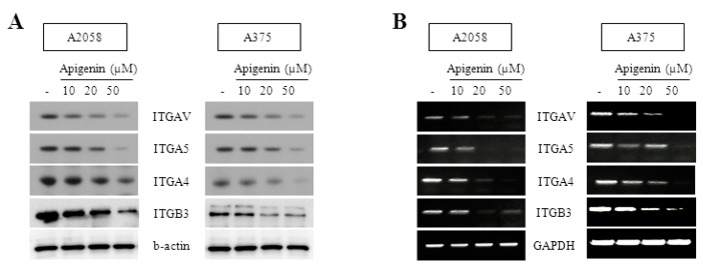
Inhibitory effect of apigenin on integrin protein expression. (**A**) mRNA expression of ITGAV, ITGA5, ITGA4 and ITGB3 in human melanoma A2058 and A375 cells. Cells were incubated with various concentrations of apigenin for 18 h. Cells were harvested and ITGAV, ITGA5, ITGA4 and ITGB3 mRNA levels were analyzed by RT-PCR; (**B**) Protein expression of ITGAV, ITGA5, ITGA4 and ITGB3 in Human melanoma A2058 and A375 cells. Cells were incubated with various concentrations of apigenin for 18 h. Cells were harvested and ITGAV, ITGA5, ITGA4 and ITGB3 protein levels were analyzed by western blotting. RT-PCR and western blotting were performed in triplicate and data are representative of three separate experiments.

According to several studies, the overexpression of integrins might be directly related to the progression of various types of malignant tumors [30,31]. Integrins are implicated in cellular migration. We investigated the expression of the integrin subunits α4, α5, αV, and β3 in A2058 and A375 melanoma cells to show the relation between altered cell migration and alterations in integrin expression. Western blot and RT-PCR analysis revealed that integrin subunits α4, α5, αV, and β3 were clearly downregulated in cell lysates of melanoma cells after apigenin treatment (Figure 2).

### 2.3. Effect of Apigenin on ERK- and FAK-Dependent Mechanisms

**Figure 3:**
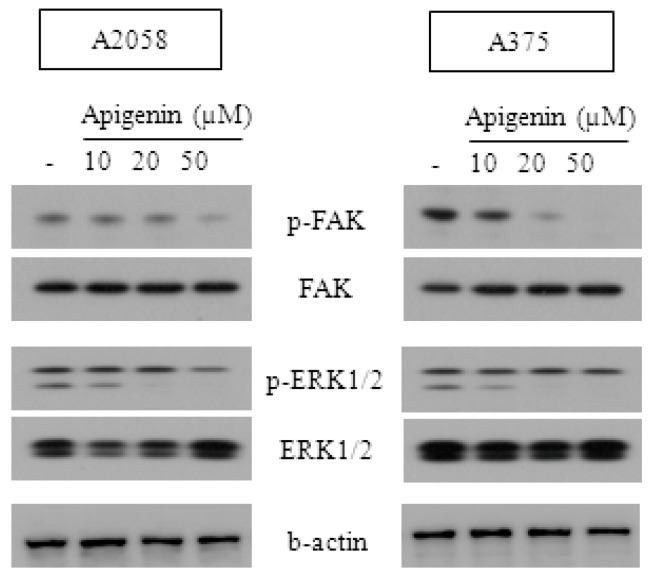
Effect of apigenin on ERK- and FAK-dependent mechanisms. A375 and A2058 melanoma cells were treated with the indicated concentrations of apigenin for 24 h. p-FAK, FAK, p-ERK 1/2 and ERK 1/2 expressions were measured by western blotting analyses. All data are representative of three separate experiments.

p-ERK1/2 is the activated form of ERK1/2. To investigate the roles of ERK1/2 in the suppression of melanoma cells, we collected the apigenin-treated cell lysates of A2058 and A375 cells and then quantified p-ERK1/2 and ERK1/2 by western blotting. As shown in Figure 3, apigenin induced a remarkable decrease in phosphorylated ERK in both cell lines, but the expression of total ERK remained unchanged. These findings indicate that apigenin can suppress ERK phosphorylation.

FAK is one of the downstream substrates of ERK. To evaluate the potential signaling cascade of ERK involved in the apigenin-induced apoptosis, we investigated the inducible activation of FAK. Phosphorylation of FAK exhibits similar synchronous changes as those of p-ERK1/2 after apigenin treatment. Thus, our present findings suggest that apigenin can repress the migratory ability of melanoma cells through ERK–FAK cascades.

### 2.4. Effect of Apigenin on the Inhibition of Cell Migration

**Figure 4:**
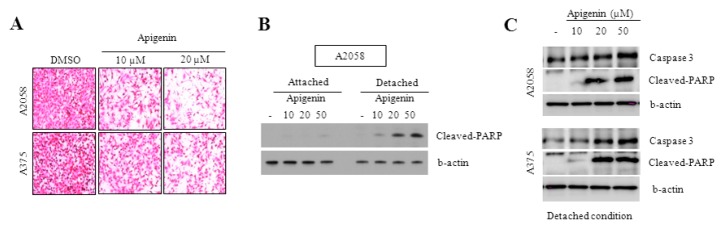
Effect of apigenin on the inhibition of cell migration. (**A**) A375 and A2058 melanoma cells migration was performed using Transwell chambers. Cells were incubated with various concentrations of apigenin for 6 h. Cell morphology was examined under a light microscope; (**B**,**C**) Caspase-3 and cleaved-PARP expressions were measured in attached and detached condition by western blotting analyses. All data are representative of three separate experiments.

The effect of apigenin on cell migration was analyzed by the Boyden chamber assay. Melanoma cell lines A2058 and A375 were treated with apigenin for 24 h at concentrations of 10 and 20 μM. A significant reduction in cell migration was noted in both melanoma cell lines as compared with untreated cells. As shown in Figure 4, the number of cells found on the lower side of the Matrigel-coated membrane filter was observed to decrease in a dose-dependent manner. Apigenin caused a dose-dependent decrease in the percentage of Transwell-migrated cells, and ~90% and ~70% inhibition of cell migration was recorded upon treatment with 20 μM of apigenin, respectively, for A2058 and A375 cells (Figure 4A). These results suggest that apigenin is highly effective in inhibiting the migration of melanoma cells.

### 2.5. Caspase-3 and PARP Cleavage Pathways Are Involved in Apigenin-Induced Anoikis

In the present study, we examined the role of caspase-3 and cleaved PARP in apoptosis. PARP helps cells to maintain their viability, and the cleavage of PARP facilitates cellular disassembly and serves as a marker of cells undergoing apoptosis [32]. The specific cleavage of PARP mediated by caspase-3 was taken as a marker of apoptosis [33,34]. Levels of caspase-3 and cleaved PARP were analyzed by western blotting in the detached condition (Figure 4B,C). Our results showed a significant dose-dependent increase in caspase-3 and cleaved PARP in melanoma cells treated with apigenin compared to the control. The data confirm published findings showing that the cleavage of caspase-3 and PARP induce apoptosis in melanoma cells [35]. Anoikis induction was associated with an increase in caspase-3 and cleaved PARP. Thus, apigenin induces anoikis by activating the caspase-3 and PARP cleavage pathways.

## 3. Discussion

Melanoma is one of the most invasive and deadly forms of skin cancer, and only a few agents are available for treating advanced stages of the disease to enable long-term patient survival. However, these agents are relatively ineffective, with overall response rates of merely 5%–20%. This underscores the need for identifying new compounds that regulate the pathways that are deregulated in melanoma [36,37].

Apigenin, a naturally occurring flavonoid phytochemical in various edible plants has been reported to possess cytotoxic effects against different cancer cells. Our results showed that apigenin exhibited dose-dependent toxicity towards A2058 and A375 cells. This observation is in agreement with the cytotoxic property of apigenin reported in other studies [16,17,38]. Three novel observations have been made in the present study. First, we found that apigenin from *C. crispus* inhibited integrin protein expression in A2058 and A375 melanoma cells. Second, we demonstrated for the first time that the downregulation of phospho FAK and phospho ERK1/2 signaling by apigenin prevented anoikis resistance in human skin cancer cells and inhibited cancer metastasis. Third, we also found that apigenin induced anoikis and inhibited migration in metastatic melanoma cells, as evidenced by cell viability assays and the detection of caspase-3 and cleaved PARP in detached cells. To our knowledge, this study is the first to directly demonstrate the anoikis-sensitizing effect of apigenin.

Integrin-mediated cell attachment has been shown to be required for cancer cell migration and metastasis [39]. In this study, we investigated the effect of apigenin on the expression of the integrin subunits α4, α5, αV, and β3 in melanoma cell lines and their contributions in the phosphorylation of FAK and ERK1/2. Previous findings showed that αV and β3 subunits of integrin have increased expression in melanoma [40]. Our results showed that apigenin dose-dependently reduced the expression of integrin subunits, leading to downstream activation of p-FAK and p-ERK 1/2 in A2058 and A375 melanoma cells. 

FAK is a cytoplasmic tyrosine kinase, which has been implicated to play a key role in integrin-mediated signal transduction pathways [41]. Constitutive activation of FAK is associated with melanoma metastasis [42]. During cell migration, FAK is most likely involved in ERK-mediated cell migration [43]. Phosphorylation of FAK stimulates a cell-signaling cascade that finally activates ERK [44]. ERK, a subfamily of MAPKs, is one of the best characterized intracellular signaling pathways, and it plays a crucial role in regulating cell migration [45]. Our present findings showed dose-dependent downregulation of the phosphorylation of FAK and ERK1/2 through apigenin treatment, which eventually induced anoikis and suppressed melanoma cell migration.

Evasion of anoikis or detachment-induced apoptosis is a key hallmark of cancer cells [46]. The overexpression of caspase-3 and cleaved PARP have a strong correlation with apoptosis of melanoma cells [35]. Caspase-3 activation subsequently leads to DNA breakage, nuclear chromatin condensation, and cell apoptosis [47]. Stimulating the expression of caspase-3 can serve as a common strategy in cancer therapies [48]. PARP, a nuclear enzyme activated during DNA damage, is known to be cleaved by caspase-3 [49]. We found that the overexpression of caspase-3 and cleaved PARP by apigenin treatment induced anoikis and suppressed the migration of melanoma cells, confirming previous findings [15,50].

## 4. Experimental Section

### 4.1. Chemicals and Reagents

Apigenin was isolated from *Carduus crispus* and kindly supplied by Dr. S.K. Kim (Konkuk University, Seoul, Korea). The isolated apigenin was subjected to various spectrometric analyses for identification. ITGAV, ITGA5, ITGA4, ITGA3, and GAPDH primers were purchased from Bioneer (Daejeon, Korea). ITGAV, ITGA5, ITGA4, ITGA3, FAK, phospho-FAK (p-FAK), ERK, phospho-ERK (p-ERK), cleaved-PARP, and β-actin antibodies were purchased from Cell Signaling Technology Inc. (Beverly, MA, USA). Agarose A was purchased from Bio Basic Inc. (Markham, ON, Canada). Dulbecco’s modified Eagle’s medium (DMEM) was obtained from Welgene Inc., (Daegu, Korea). Random oligonucleotide primers and Moloney-murine leukemia virus (M-MLV) reverse transcriptase were purchased from Promega (Madison, WI, USA). dNTP Mix and SYBR Green Ex Taq were purchased from TaKaRa (Seoul, Korea). Oligonucleotide primers were purchased from Bioneer. All other cell culture reagents and chemicals were of analytical grade and were purchased from Sigma Chemical Co. (St. Louis, MO, USA).

### 4.2. Cell Culture

Human melanoma cancer cell lines (A375 and A2058) obtained from American Type Culture Collection (Manassas, VA, USA) were maintained in DMEM supplemented with 10% fetal bovine serum (FBS), 100 U/mL penicillin, and 100 μg/mL streptomycin. Cells were incubated at 37 °C in a humidified atmosphere containing 5% CO_2_.

### 4.3. 3-(4,5-Dimethylthiazol-2-yl)-2,5-diphenyl Tetrazolium Bromide Assay

Cell viability was evaluated by 3-(4,5-dimethylthiazol-2-yl)-2,5-diphenyl tetrazolium bromide (MTT) assay as described previously [51]. Briefly, cells were seeded (5 × 10^3^ cells/well) in 96-well plates in the absence of or at varying concentrations of apigenin for different time intervals at 37 °C. The treatments were started at different concentrations 24 h after seeding. Thereafter, cells were incubated in MTT solution for 4 h, and then, formazan crystals resulting from MTT reduction were dissolved by adding 100 μL of DMSO in each well and gently shaking for 15 min. The absorbance of cultures was measured using a multiwell spectrophotometer at a test wavelength of 560 nm. Results were calculated as percentage of absorbance in control cultures.

### 4.4. Analysis of Cell Morphology

A375 and A2058 cells were placed in 6-well culture plates (5 × 10^4^ cells/mL) and allowed to attach for 8 h. Next, the cells were left untreated or were treated with apigenin at different concentrations. After incubation for 24 h, morphological changes of the cells were observed by light microscopy and photographed at a magnification of 100×.

### 4.5. Preparation of Total Cell Extracts and Immunoblot Analysis

For western blot analysis, whole-cell extracts were prepared by adding RIPA buffer (Sigma-Aldrich) containing a protease inhibitor cocktail. Protein concentrations of all samples were determined by using Coomassie Plus (Bradford) protein assay reagent (Thermo Scientific, Rockford, IL, USA). Extracts were subjected to SDS-polyacrylamide gel electrophoresis and transferred to nitrocellulose membranes. The membranes were probed with primary antibodies, followed by incubation with peroxidase-conjugated secondary antibodies (Danvers, MA, USA). Next, proteins of interest were visualized using enhanced chemiluminescence. Images were captured by a Fujifilm LAS-3000 system and the LAS-2000 software (Fujifilm, Tokyo, Japan).

### 4.6. RNA Isolation and RT-PCR

Total RNA was extracted from melanoma cells with or without apigenin treatment using the Trizol reagent (Invitrogen, Waltham, MA, USA) according to the manufacturer’s instructions. Total RNA (5 µg) was reverse transcribed to cDNA with 1 µM oligo(dT)_15_, 500 µM of dNTP, 0.05 M Tris-HCl, pH 8.3, 0.075 M KCl, 0.003 M MgCl_2_, RNase inhibitor (1 unit/µL), and M-MLV reverse transcriptase (10 unit/µL) at 42 °C for 1 h. Polymerase chain reaction (PCR) was performed in reaction buffer [cDNA, 1.25 U Taq DNA polymerase (Promega), 3′- and 5′-primers (50 μM each), and 200 mM dNTP in 200 mM Tris-HCl buffer (pH 8.4) containing 500 mM KCl and 1–4 mM MgCl_2_]. Thermal cycling conditions were as follows: 15-s denaturation at 94 °C, 30-s annealing at 55 °C, and 1-min extension at 72 °C. Synthesized cDNA was subjected to amplification on an ABI 7500 real-time PCR platform using the SYBR Green System (Applied Biosystems, Foster City, CA, USA). Gene-specific primer sequences used for amplification were as follows: ITGAV (forward, 5′-AAT CTT CCA ATT GAG GAT ATC AC-3′; reverse, 5′-AAA ACA GCC AGT AGC AAC AAT-3′), ITGA5 (forward, 5′-TGC AGT GTG AGG CTG TGT ACA-3′; reverse, 5′-GTG GCC ACC TGA CGC TCT-3′), ITGA4 (forward, 5′-GCT TCT CAG ATC TGC TCG TG-3′; reverse, 5′-GTC ACT TCC AAC GAG GTT TG-3′), ITGB3 (forward, 5′-CCG TGA CGA GAT TGA GTC A-3′; reverse, 5′-AGG ATG GAC TTT CCA CTA GAA-3′), and GAPDH (forward, 5′-GCA AAG TGG AGA TTG TTG CCA TC-3′; reverse, 5′-CAT ATT TCT CGT GGT TCA CAC CC-3′). After amplification, PCR products were electrophoresed in 2% agarose gels (Invitrogen) containing 2.0 µg/mL ethidium bromide.

### 4.7. Cell Migration Assay

Cancer cell migration assays were performed using Transwell chambers purchased from Sigma-Aldrich. For the migration assay, A375 and A2058 melanoma cells in 0.1 mL of FBS-free medium were seeded in the upper chamber. The lower chamber was filled with complete culture medium as a chemotactic agent, and cells were then incubated for 6 h. Cells that migrated were then stained with hematoxylin and eosin. The filter membranes, containing the migrating cells, were then placed on a glass slide and analyzed using an Olympus IX51 microscope (Olympus, Tokyo, Japan).

### 4.8. Statistical Analysis

All assays were performed in at least three independent experiments. The mean ± SD values were determined, and statistical comparisons between groups were performed using one-way ANOVA followed by Tukey’s multiple comparison test. All statistical analyses were performed with the Prism 5 software (GraphPad Software Inc., San Diego, CA, USA). *p* values of ≤0.05 were considered statistically significant.

## 5. Conclusions

In conclusion, we have demonstrated that apigenin significantly inhibited the growth of melanoma cells and induced apoptosis. We herein report for the first time the induction of anoikis by apigenin in human skin cancer cells. One plausible mechanism of action might be partly mediated by the modulation of FAK/ERK, caspase-3, cleaved PARP, and integrin signaling pathways. Taken together, our results suggest that apigenin might be a promising anticancer agent with potential applications in the treatment of skin cancer.
